# Geriatrics Education Mentor (GEM) Program: Outcomes from Pairing Students and Older Adults During Medical School

**DOI:** 10.1093/geroni/igaf122.3267

**Published:** 2025-12-31

**Authors:** Hannah Griffin, David Wihry, Marilyn Gugliucci

**Affiliations:** University of New England College of Osteopathic Medicine, Biddeford, Maine, United States; University of Maine, Bangor, Maine, United States; University of New England College of Osteopathic Medicine, Kennebunk, Maine, United States

## Abstract

Currently, only 15% of medical schools have mentor programs that educate students to work with older adults and their health. The University of New England College of Osteopathic Medicine Geriatrics Education Mentor (GEM) program is an experiential education component within the 1st and 2nd year curriculum whereby students and older adults are paired to conduct five “home-visit” and complete geriatrics specific assignments. A pre/post survey design assessed students’ competence and confidence with older people (assigned GEMs). The survey was emailed to first-year medical students (avg. 174/class) before they began GEMs and was sent again upon completion of the program at the end of their second year during academic years 2020-2021 through 2023-2024. Data were analyzed in RStudio. Results had a 26% response rate that included overall data (N = 133/Pre & N = 138/ post), 24 responses were matched assessments. No significant change in students’ interest (p > 0.4), perceived readiness and competence (p > 0.07), or confidence in working with older adults (p > 0.5) was revealed. However, perceived readiness and confidence increased from pre- to post-test, indicating that a larger sample size might reveal significance. Self-reported knowledge of older adult healthcare needs increased, pre- to post-test, with the paired subset showing a significant increase (p < 0.015). Although a small sample, these findings provide a foundation for medical schools to consider implementing a mentor type program to augment medical student skill/knowledge in older adult health care to advance age-friendly health care.

